# Spatial distribution of the risk of dengue fever in southeast Brazil, 2006-2007

**DOI:** 10.1186/1471-2458-11-355

**Published:** 2011-05-20

**Authors:** Ricardo Cordeiro, Maria R Donalisio, Valmir R Andrade, Ana CN Mafra, Luciana B Nucci, John C Brown, Celso Stephan

**Affiliations:** 1 Department of Social and Preventive Medicine, School of Medical Sciences, State University of Campinas, São Paulo, Brazil; 2 State Health Department of São Paulo - Superintendence for Control of Endemic Diseases SUCEN, Campinas, São Paulo, Brazil; 3 Department of Geography, University of Kansas, Lawrence, KS, USA

## Abstract

**Background:**

Many factors have been associated with circulation of the dengue fever virus and vector, although the dynamics of transmission are not yet fully understood. The aim of this work is to estimate the spatial distribution of the risk of dengue fever in an area of continuous dengue occurrence.

**Methods:**

This is a spatial population-based case-control study that analyzed 538 cases and 727 controls in one district of the municipality of Campinas, São Paulo, Brazil, from 2006-2007, considering socio-demographic, ecological, case severity, and household infestation variables. Information was collected by in-home interviews and inspection of living conditions in and around the homes studied. Cases were classified as mild or severe according to clinical data, and they were compared with controls through a multinomial logistic model. A generalized additive model was used in order to include space in a non-parametric fashion with cubic smoothing splines.

**Results:**

Variables associated with increased incidence of all dengue cases in the multiple binomial regression model were: higher larval density (odds ratio (OR) = 2.3 (95%CI: 2.0-2.7)), reports of mosquito bites during the day (OR = 1.8 (95%CI: 1.4-2.4)), the practice of water storage at home (OR = 2.5 (95%CI: 1.4, 4.3)), low frequency of garbage collection (OR = 2.6 (95%CI: 1.6-4.5)) and lack of basic sanitation (OR = 2.9 (95%CI: 1.8-4.9)). Staying at home during the day was protective against the disease (OR = 0.5 (95%CI: 0.3-0.6)). When cases were analyzed by categories (mild and severe) in the multinomial model, age and number of breeding sites more than 10 were significant only for the occurrence of severe cases (OR = 0.97, (95%CI: 0.96-0.99) and OR = 2.1 (95%CI: 1.2-3.5), respectively. Spatial distribution of risks of mild and severe dengue fever differed from each other in the 2006/2007 epidemic, in the study area.

**Conclusions:**

Age and presence of more than 10 breeding sites were significant only for severe cases. Other predictors of mild and severe cases were similar in the multiple models. The analyses of multinomial models and spatial distribution maps of dengue fever probabilities suggest an area-specific epidemic with varying clinical and demographic characteristics.

## Background

Dengue fever is an infectious, epidemic disease, transmitted by the vector *Aedes aegypti*. The disease is becoming endemic mainly in tropical regions, where the expansion of urban populations, impoverished and crowded areas, and poor infrastructure create ideal habitats for vector proliferation and consequent spread of the virus. Dengue incidence is seasonal, increasing during months of highest temperature and precipitation. The disease in Brazil is mostly found in the southeast region. In 2008, however, the presence of three virus serotypes was recorded (Den1, Den2 and Den3) in most Brazilian states [1].

Vector control measures, passive epidemiologic surveillance, and educational campaigns have not been very effective in most Brazilian municipalities, highlighting the complexity of the disease and the difficulty of controlling it. Many factors have been associated with circulation of the virus and vector, although the dynamics of transmission are not yet fully understood [2]. Some authors point out that poverty, household water supply source, conditions of drainage disposal and garbage collection would be associated with the occurrence of the vector and of the disease [3-5]. Others have mentioned low education and income levels and high population density as determinants of the disease [4,6,7]. In other studies, movements of people and goods, linked with migration and trade patterns, have been identified as indicators of risk of infestation by the vector and virus transmission [8].

In the city of Campinas, state of São Paulo, the number of identified vector foci has increased since 1987, and the autochthonous transmission of the disease was first recorded in 1996 and 1997. Large epidemics occurred in the city in 1997-1998, 2002-2003 and 2006-2007, with laboratory-confirmed incidence of 113, 151, and 669 cases per 100,000 inhabitants, respectively.

The understanding of the dynamics of the epidemic in Brazil has expanded through the use of tools traditionally used in geography and cartography, namely geospatial data analysis. Most of these studies take a descriptive ecological approach and analyze the disease risk (incidence) within specific geographic areas [2,4,9,10]. Few studies focus on disease risk in urban spaces using spatial point pattern analysis, which generates more precise information about risk factors related to transmission in different epidemic contexts [11-14].

The objective of the present study was to estimate the spatial distribution of the risk of occurrence of dengue fever in an area of the municipality of Campinas, in the years 2006 and 2007, considering socio-demographic, ecological, case severity, and household infestation variables. It was also assessed non-spatial predictors of mild and severe dengue infection.

## Methods

### Study design

This is a population-based spatial case-control study with multinomial response in which the independent variable of major interest was geographical location of residence. The population source for the cases in this study was all individuals, aged 20 years or above, who lived in the South District of Campinas during the study. The criteria for inclusion as a case were: belonging to the source population and being a laboratory-confirmed autochthonous case of dengue fever, diagnosed between October 2006 and September 2007. Patients enrolled in the study were diagnosed by municipal health service teams after clinical and laboratory confirmation carried out the official state laboratory. All patients with laboratory confirmation of dengue were contacted for an interview and invited to participate in the study.

Controls were matched with cases within a timeframe through a random two-stage sample. That is, when a case was enrolled in the study, it was matched with a random selection of a control household. The case to control ratio was 1:1. In the first sample stage, households were drawn from a universal record of South District households, maintained by healthcare centers, and then each household was visited. Households were eliminated from the sample, without replacement, if no one was home over three consecutive visits on different days. In the second sample stage, after classifying household occupants, an individual was randomly chosen from each household to participate in the study. Controls were not tested for dengue infection.

### Data collection

Data were collected from October 2006 to September 2007 through home interviews and inspection of living conditions in and outside the home in both the case and control groups in the South District of Campinas, a city located in the southeast region of the State of São Paulo, roughly 100 km from the capital, with a population of 969,396 inhabitants in 2000. Campinas is an important center of high-tech industrial development; it has Brazil's third largest industrial concentration, with comparatively high economic indicators and living conditions. Though it is a region of relative economic wealth and development, the municipality suffers severe social problems such as high rates of violence and unemployment. The South District of Campinas has a population of approximately 270,000 inhabitants, encompassing 17 Health Centers. Since 1999, this district experiences the highest incidence of dengue fever in Campinas.

### Study variables

All participants in the research gave informed written consent after a full explanation of the study; the institutional ethics committee of the School of Medical Sciences of State University of Campinas, São Paulo, approved the study. During household visits, a trained team used a structured interview to obtain the following information about all participants: age (in years), gender, education level of head of household (years of schooling), whether or not they stay at home during the work day (yes/no), whether or not they spend any period of the day outside the neighborhood (out of the District), working or studying (yes/no), previous report of dengue fever (self-reported yes/no), occupation, work and school addresses. Additional information about the household was also obtained during the interview and referred to conditions within the preceding year: family income, household water delivery (centralized, well, water truck or "other"), experience with water shortages (yes/no/sometimes), whether household members practice water storage or not (yes/no), pronounced presence of garbage immediately surrounding the home (yes/no), garbage collection frequency (times per week), household experience with floods (yes/no), reports of mosquito bites during the day (yes/no), basic sanitation (indoor plumbing and centralized treatment/cesspool/open air), occurrence of mosquito breeding sites (yes/no), number of breeding sites, presence of larvae (yes/no) and home address.

During the visit, an entomological survey was carried out in the household and surroundings. Assessments of larvae densities (number of breeding sites for positive *Aedes aegypti *per 100 households) were also analyzed. This is the larva indicator that has been used as a parameter in most of the dengue fever control programs in the country [15]. Household data quality was assured by randomly choosing 10% of the sample sites and repeating the entire survey, correcting any errors detected. It was possible to correct 17 (1.3%) records (6 cases and 11 controls). For each of the dengue fever cases, the following information was obtained from the reporting form on signs and symptoms from the health services center: disease development, occurrence of bleeding and hospitalization.

Clinical data were used for classifying dengue fever cases according to severity into two groups: *mild dengue fever *(dengue fever without warning signs, without spontaneous or induced bleeding by the tourniquet test) and *severe dengue fever *(dengue fever with warning signs and/or positive tourniquet test and/or bleeding and/or hypovolemic shock and/or any other signs of severity).

Geographical coordinates of addresses of cases and controls were obtained directly in the field, using a portable Global Positioning System (GPS) with an average accuracy of 7 meters. Geographical coordinates of residence of cases and controls were identified on a digital cartographic database of the study area (UTM 23 S projection, SAD 69 datum), containing, among other information, streets, blocks and Health Districts. The database was provided by the Health Department of the Campinas City Hall.

### Statistical analysis

A simple logistic binomial model was initially adjusted, with the status of the individual as the response variable (case or control), and the covariates of interest to the study, one by one, as predictor variables. Based on these simple models, a multiple binomial model was adjusted, with the status of the individual as the response variable and containing as predictor variables those whose *P *values of effect estimates were less than 0.25 in the simple adjustments, and which, after multiple adjustments, had a *P *value below 0.05.

To assess the spatial variability of the probability of disease, nominal multinomial response estimates were calculated, considering the classification of individuals in three response categories (control, mild cases and severe cases). The response variable was represented by *Y *and will take values *y *= 1 (mild dengue fever), y = 2 (severe dengue fever), and y = 3 (controls).

Let **x **be a matrix with geographic coordinates that maps individuals in the South District of Campinas (herein represented by ℜ) and **z **be a matrix of covariates of interest associated with individuals studied.

The probability that any individual in the data set belongs to category *i *is given by:(1)

In which i = 1 refers to mild cases, i = 2 refers to severe cases, i = 3 refers to controls, *λ*_*i*_(**x,z**) is the spatial density function of occurrences of type *i *in ℜ (number of occurrences per area unit in **x **location),  and *qi *is the ratio between the proportion of people in category *i *included in the sample and the proportion on people in category *i *in the source population of cases.

Analyses were performed with generalized additive models [16] estimating a semi-parametric model where covariates of matrix **z **were adjusted in a parametric manner and the spatial portion (referring to matrix **x**) was adjusted in a non-parametric way with cubic smoothing splines using three degrees of freedom each smoothing.

We adjusted a multinomial logistic regression [17], that can be represented as:(2)

where *f*() is the smooth non-parametric function. Odds Ratio of each parametric variable were obtained from multinomial logistic regression.

The spatial distribution of mild and severe dengue fever risk, herein represented by the conditional probability of disease given the exposure, was obtained by probability estimates described in equation 2, in each one of the two comparisons.

Models were adjusted by R2.7 software (http://www.r-project.org accessed on 29/03/2011) through the vgam function of the VGAM package [18]. The map was plotted on a grid with 200 × 200 points.

Monte Carlo simulations were used to define non-significant areas with 95% confidence according to a normal distribution of 300 non-parametric estimates [19]. The result of these simulations is presented in the figures referring to the estimated conditional probability. A dotted texture was used to identify non-significant areas.

## Results

The sample was composed of 555 cases and 736 controls. Due to missing data, only 538 cases and 727 controls (98%) were analyzed, 377 of which were mild cases and 161 severe cases.

Table 1 shows characteristics of study population. There was a high proportion of poor sanitation and garbage around the houses in both cases and control groups. The presence of mosquito biting during the day (62.2%) and breeding sites around the houses (7.7%) was high, especially among cases group.

**Table 1:** Average (standard deviation) and percentage of variable categories among dengue fever cases and controls, Campinas, 2007

Variable	Cases (n = 538)	Controls (n = 727)	Total (n = 1265)
Age, mean years (SD)	40.8 (13.9)	45.6 (15.4)	43.6 (14.9)
Sex:			
Male	45%	44.8%	44.9%
Female	55%	55.2%	55.1%
Education level, head of household, mean years of study (SD)	6.7 (4.1)	7.9 (4.5)	7.4 (4.4)
Stays home during the day:			
Yes	36.6%	49.9%	44.3%
No	63.4%	50.1%	55.7%
Has had dengue fever:			
Yes	2.6%	2.7%	2.7%
No	97.4%	97.3%	97.3%
Water supply:			
Centralized	92.6%	98.2%	95.8%
Well, Truck or "Improvised"	7.4%	1.8%	4.2%
Water shortage:			
Yes	6.1%	5.5%	5.8%
No	77.5%	77.3%	77.4%
Sometimes	16.4%	17.2%	16.8%
Water storage:			
Yes	14.1%	3.6%	8.1%
No	85.9%	96.4%	91.9%
Garbage around the house:			
Yes	33.5%	15.7%	23.2%
No	66.5%	84.3%	76.8%
Garbage collection (times per week):			
≤2	77.7%	95.2%	87.7%
>2	22.3%	4.8%	12.3%
Flood:			
Yes	27.5%	7.3%	15.9%
No	72.5%	92.7%	84.1%
Mosquito bites during the day:			
Yes	67.8%	58.1%	62.2%
No	32.2%	41.9%	37.8%
Basic sanitation:			
Centralized	56.9%	81.8%	71.2%
Cesspool	18.8%	13.3%	15.7%
Open air	24.3%	4.8%	13.1%
Presence of breeding sites:			4
Yes	61.9%	37.3%	7.7%
No	38.1%	62.7%	52.3%
Number of breeding sites:			
0	38.1%	62.7%	52.3%
1 to 10	31.4%	18.6%	24.0%
>10	30.5%	18.7%	23.7%
Presence of Larvae:			
Yes	1.5%	1.4%	1.4%
No	98.5%	98.6%	98.6%
Assessment of Larvae Density^a^, mean (SD)	1.6 (0.8)	0.9 (0.9)	1.2 (0.9)

^a ^Assessment of Larvae Density = number of positive breeding sites per 100 domiciles

**Figure 1:**
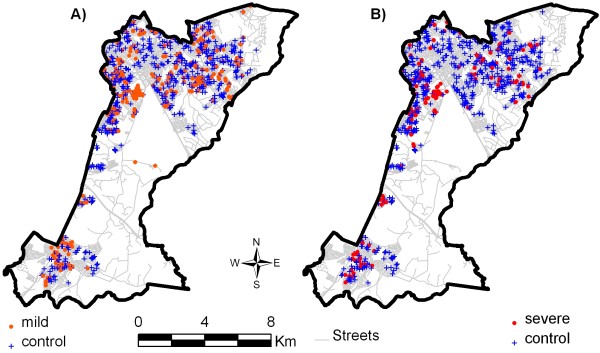
**Spatial distribution of points referring to the sample of dengue fever, Campinas, SP, 2007**. A) Mild cases of dengue fever (377) and Controls (727). B) Severe cases (161) and Controls (727).

The spatial distribution of these points is represented in Figures 1a and 1b.

The results of simple and multiple binomial models, that is, considering all cases as a single category, are shown in Table 2. Age, staying home during the day, storing water, basic sanitation, garbage collection, number of breeding sites found, and larvae density were statistically associated with the occurrence of dengue fever in the South District.

**Table 2:** Simple and multiple analyses (binomial response) of dengue fever cases and controls, Campinas, SP, 2007

Variable	**Crude OR**^**a **^	95% CI	**Adjusted OR**^**a **^	95% CI
Age (years)	0.98	0.97, 0.99	0.99	0.98, 1.00
Sex				
Male	1.01	0.80, 1.26		
Female	-			
Education level, head of household (years of study)	0.94	0.91, 0.96		
Stays home during the day				
Yes	0.58	0.46, 0.73	0.45	0.34, 0.60
No	-		-	
Has had dengue fever				
Yes	0.94	0.47, 1.89		
No	-			
Water supply				
Centralized	-			
Well, Truck or "Improvised"	4.41	2.34, 8.33		
Water shortage				
Yes	1.11	0.69, 1.79		
No	-			
Water storage				
Yes	4.44	2.80, 7.03	2.48	1.41, 4.35
No	-		-	
Garbage around the house				
Yes	2.70	2.07, 3.54		
No	-			
Garbage collection (times per week)				
≤ 2	5.68	3.82, 8.43	2.64	1.56, 4.47
> 2	-	-		
Flood				
Yes	4.83	3.43, 6.76		
No	-			
Mosquito bites during the day				
Yes	1.52	1.21, 1.93	1.82	1.36, 2.44
No	-		-	
Basic sanitation				
Centralized	-		-	
Cesspool	2.02	1.48, 2.76	1.19	0.80, 1.79
Open air	7.28	4.89, 10.8	2.94	1.77, 4.87
Presence of breeding sites				
Yes	2.73	2.17, 3.44		
No	-			
Number of breeding sites				
0	-		-	
1 to 10	2.78	2.10, 3.68	1.83	1.31, 2.54
>10	2.68	2.03, 3.55	1.60	1.12, 2.27
Presence of Larvae				
Yes	1.08	0.42, 2.76		
No	-			
Assessment of Larvae Density ^b^	2.62	2.25, 3.04	2.30	1.96, 2.70

^a ^OR = odds ratio

^b ^Assessment of Larvae Density = number of positive breeding sites per 100 domiciles

By adjusting the multiple multinomial model we obtained the statistically significant variables associated with the occurrence of mild and severe cases, shown in Table 3. Age and number of breeding sites greater than 10 were not significant factors for the occurrence of mild cases; however they were relevant for severe cases. Staying home during the day was a protective factor for the disease both for severe and mild cases. Variables related to the presence of the vector - the practice of water storage in the home, greater larvae density, number of breeding sites, and reports of mosquito bites during the day were associated with a higher occurrence of severe and mild dengue fever (Table 3).

**Table 3:** Multiple analysis (multinomial response) in mild and severe cases of dengue fever, both compared with controls, Campinas, SP, 2007

Variable	**OR**^**a **^**Mild cases**	95%CI	**OR**^**a **^**Severe cases**	95%CI
Age (years)	0.99	0.98, 1.00	0.97	0.96, 0.99
Stays home during the day				
Yes	0.47	0.35, 0.64	0.38	0.24, 0.58
No	-		-	
Water storage				
Yes	2.39	1.33, 4.31	2.75	1.37, 5.53
No	-		-	
Garbage collection (times per week)				
≤2	2.78	1.61, 4.81	2.30	1.16, 4.56
> 2	-		-	
Mosquito bites during the day				
Yes	1.68	1.23, 2.29	2.34	1.50, 3.64
No	-		-	
Basic sanitation				
Centralized	-		-	
Cesspool	1.26	0.82, 1.95	1.03	0.56, 1.89
Number of breeding sites				
0	-		-	
1 to 10	1.76	1.24, 2.50	2.05	1.25, 3.34
>10	1.46	1.00, 2.13	2.06	1.23, 3.45
Assessment of Larvae Density ^b^	2.13	1.80, 2.54	2.86	2.25, 3.64

^a ^OR = odds ratio

^b ^Assessment of Larvae Density = number of positive breeding sites per 100 households.

**Figure 2:**
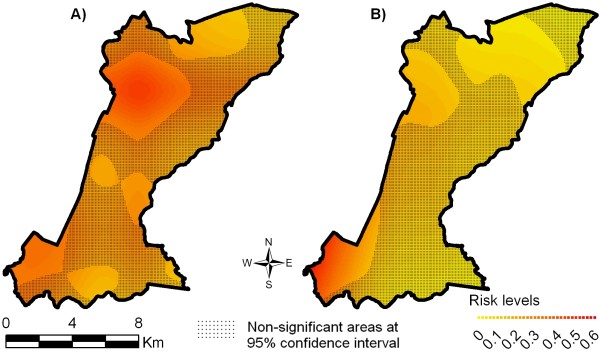
**Spatial distribution of dengue fever risk obtained through the multinomial model, Campinas, SP, 2007**. Responses are: severe cases, mild cases and controls. N = 1265 (Figure estimated without covariates). A) Risk of mild dengue fever. B) Risk of severe dengue fever.

**Figure 3:**
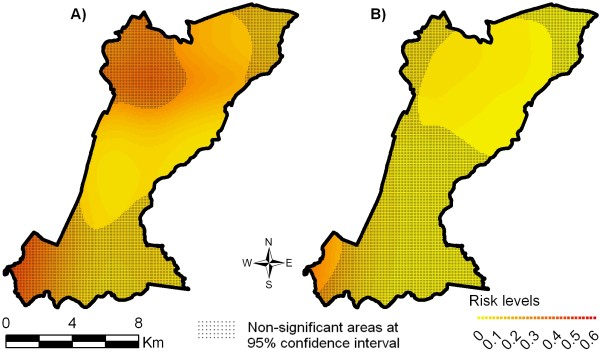
**Adjusted spatial distribution of dengue fever risk obtained through the multiple multinomial model, Campinas, SP, 2007**. Response: severe cases, mild cases, and controls. N = 1,265 (Figure estimated with covariates). A) Risk of mild dengue fever. B) Risk of severe dengue fever.

The results of crude and semi-parametric spatial analyses identified different transmission patterns of mild and severe dengue fever in the urban space. The deviance between these models was 459.368 with 140.147 degrees of freedom, indicating that the inclusion of non-spatial co-variables was statistically significant (p-value < 0.0001 for Chi-square test). Figures 2a and 2b show the crude spatial distribution of the risk of mild and severe dengue fever, respectively, in the South District of Campinas and significant areas in the model. Figures 3a and 3b show the semi-parametric spatial distribution of the risk of mild and severe dengue fever, i.e. controlling for the non-spatial covariates selected in the multinomial adjustment.

Considering only the areas where the population is present (Figure 1) and the areas with significant estimations (non-hatched), the predicted probabilities of mild dengue fever (Figure 2a) was up to 0.4 in the southeast and up to 0.3 in the northwest, in addition to an area of lower risk in the north, 0.2. When parametric covariates were included in the model (Figure 3a), these probability areas loose their significance and only the area in the north remained with a probability of 0.15 to 0.2 for mild cases.

For severe dengue fever, for the crude spatial model (Figure 3a), there was a probability of 0.3 to 0.4 in the southeast, of 0.15 in the northwest and still a small chance of occurrence of severe cases in the north of the study region (risk = 0.05 to 0.09). When covariates were incorporated (semi-parametric model, Figure 3b), they generated a map where the probability of occurrence of more severe cases of dengue fever was limited to the northwest (ranging from 0.05 to 0.07) and practically disappeared in the southeast.

## Discussion

The variables associated with the risk of dengue fever in this study reinforce previous concerns with socio-environmental, demographic and entomological factors, which have also been the focus of disease control programs in many parts of the world [8]. Some of the variables analyzed are indirect markers of the socioeconomic conditions of the population and, in this study, were found to be risk predictors for dengue fever both in mild and severe forms. These conditions include the lack of access to basic sanitation (open air sewage), garbage collection less than twice a week, and irregular water supply. Higher dengue seroprevalence in more deprived neighborhoods has been found in several Brazilian cities, especially in particularly crowed areas [9,11,12]. The lower weekly frequency of garbage collection, in addition to being a social marker and an indicator of access to sanitation services, may be related to an increase in favorable vector breeding sites in areas close to homes. The type of household water source, and particularly the practice of storing water due to intermittent supply in dry periods, has been associated with the proliferation of the vector and a determining factor for the transmission of dengue fever in many studies [20,21].

The multiple regression model used in this study showed relationships between variables related to mosquito breeding sites and the transmission of dengue fever, including both mild and severe cases. This result suggests that staying home during the day was a protective factor, calling attention to the importance of dengue transmission at work, schools and other public spaces. The number of breeding sites, reports of mosquito bites during the day, and larvae density have been identified as indicators of the transmission of dengue fever in most medium and large cities and metropolitan regions in Brazil [22,23]. In a study of seroprevalence and the density of vectors in Rio de Janeiro, authors identified viral transmission occurring in places where there is the greatest movement of people, such as public spaces, bus stations, and commercial centers [11].

This study showed that the spatial distribution of risks of mild and severe dengue fever differed from each other in the 2006/2007 epidemic, in the study area. This pattern could only be identified through the use of multinomial analysis. When cases and controls were analyzed spatially only, i.e. without non-spatial covariates, the regions more likely to have mild cases could be clearly seen, especially in the northwest and also southeast. As to severe cases, they were identified most often in the southwest and less in the northwest. No epidemiological explanation for the case generation process can be inferred based on these maps; their importance is to reveal the areas with higher crude probability for the disease.

Visual comparison suggests that spatial analysis of risk of mild and severe dengue fever differed from each other in the maps (Figures 2 and 3). These differences, both in terms of amplitude of the area and in probability values, suggest different disease dissemination mechanisms with different clinical patterns. The use of the multinomial model proved to be an important tool for this analysis. The comparison of Figures 2a and 2b, and 3a and 3b highlights the differences between the crude model and the semi-parametric model. The inclusion of non-spatial covariates smoothes the risk distribution as well as reduces its statistical significance. They provide evidence that in adjusted models, socio-environmental, sanitation, and entomological covariates explain most of the production of dengue fever cases in this population, through their spatial distribution.

The records of dengue fever circulation in this same region during the 2002/2003 epidemic, in Campinas, show that the southwest region was affected more intensely, which leads to the suspicion of previous infections, thus worsening the clinical manifestation of dengue fever. This trend has been observed in many regions of the world, explaining the occurrence of hemorrhagic dengue fever epidemics [24]. It is worth mentioning that determining the immunity status of individuals as a result of previous infections and including this in our analysis was beyond the scope of this research, and there is no information about the circulation of other strains of the virus in the region. These variables could contribute to the understanding of the transmission dynamics, in addition to explaining the infection of different age groups and clinical features of the disease in the District. Figures 2 and 3 should be compared with Figure 1, in order to assess the "edge effects" of interpolating spatial data in restricted geographic areas. In this case, it appears that darker significant risk areas at the southwestern border of study area (Figure 2) refer to cases occurrence not exactly at the border. These effects were not controlled in the model.

One limitation of the study is the lack of serologic tests in the control population leading to potential selection bias, as controls could have had asymptomatic dengue infection or dengue in the past. This potential bias could decrease the association between variables and dengue occurrence toward the null hypothesis i.e. dengue cases and controls could be exposed to the same predictors variables, weakening statistical significance in the model.

## Conclusions

The analyses of multinomial models and spatial distribution maps of dengue fever probabilities suggest a sector-specific epidemic with different clinical and demographic characteristics. Risk areas of mild and severe cases were not coincident on the maps. Age and presence of more than 10 breeding sites were significant only for severe cases. Other predictors of mild and severe cases were similar in the multiple models as discussed above. Results can contribute to a better understanding of dengue epidemics among researchers and practitioners. Cartography is an important tool to identify sites in densely populated cities where the risk for contracting dengue fever is greatest. Moreover, the use of generalized additive models and multinomial logistic analysis may help identify specific spatial transmission patterns. The spatial component of transmission can be isolated, after controlling for variables of interest, thus contributing to future studies that consider new hypotheses and variables in the analysis.

## Competing interests

The authors declare that they have no competing interests.

## Authors' contributions

RC proposed, coordinated the project and participated in the design and analysis of results; MRD participated in the proposal and in elaboration of the manuscript; VRA carried out the field research as part of his doctoral dissertation; ACNM, LBN and CS participated on statistical and geospatial analysis. JCB participated in the revision of the manuscript. All authors participated in discussion of results, read and approved the final manuscript.

## Pre-publication history

The pre-publication history for this paper can be accessed here:

http://www.biomedcentral.com/1471-2458/11/355/prepub
